# Editorial: Tumor immune microenvironment topographies for prediction and evaluation: unlock the mystery of the therapeutic effects and adverse events of tumor immunotherapy

**DOI:** 10.3389/fonc.2023.1301340

**Published:** 2023-11-01

**Authors:** Xiaoran Yin, Yan Feng, Bowen Zhang, Xueyan Mao, Siying Chen, Yuyan Wang, Sidney W. Fu

**Affiliations:** ^1^ Department of Oncology, The Second Affiliated Hospital of Xi’an Jiaotong University, Xi’an, Shaanxi, China; ^2^ Department of Gastroenterology, The First Affiliated Hospital of Xi’an Jiaotong University, Xi’an, Shaanxi, China; ^3^ Department of Medical Intensive Care Unit, The First Affiliated Hospital, Sun Yat-sen University, Guangzhou, Guangdong, China; ^4^ Department of Pharmacy, The First Affiliated Hospital of Xi’an Jiaotong University, Xi’an, Shaanxi, China; ^5^ Key Laboratory of Carcinogenesis and Translational Research (Ministry of Education/Beijing), Department of Thoracic Medical Oncology, Peking University Cancer Hospital & Institute, Beijing, China; ^6^ Division of Cancer Prevention (Cancer Biomarkers Research Group), National Cancer Institute, Rockville, MD, United States

**Keywords:** tumor microenvironment, immune landscape, computational technology, tumor immunotherapy, prognostic model

The tumor microenvironment (TME) constitutes a dynamic system comprising tumor cells, stromal cells and inflammatory cells, among others. Within this milieu, tumor cells adapt, exerting profound influence on antitumor immunity, drug resistance, metastasis, and immune evasion. Immunotherapy harnesses the host’s immune system to prevent and combat tumors. Although immunotherapy has achieved notable success, some patients exhibit resistance to treatment, and the mechanisms are poorly elucidated. Confronting these challenges represents a formidable undertaking that requires a deeper understanding of the intricate interplay among these components. The potential for enhancing immunotherapy and reshaping clinical outcomes in pan-tumor therapies lies in the remodeling and recognition of individual TME characteristics (**Figure 1**).

**Figure 1:**
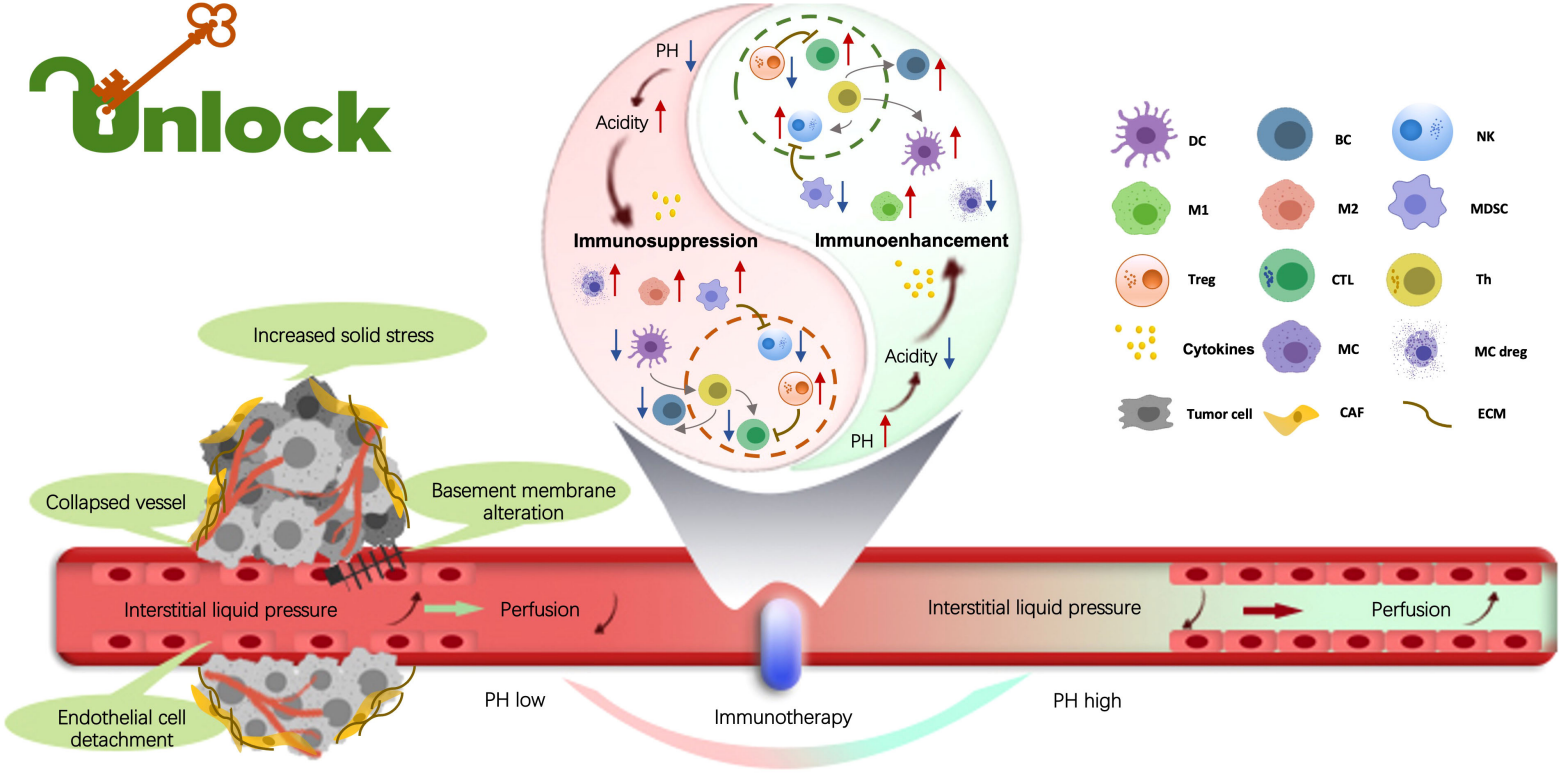
Within the tumor microenvironment (TME), the biological, chemical, and mechanical factors influence tumor promotion, metastasis, drug resistance, and immune evasion. Tumor-infiltrating immune cells (TIICs) play the crucial role in regulating these processes. By exploring the distinct attributes of TIICs, the topic offers the potential to unveil novel approaches and cutting-edge technologies, driving the advancement of tumor immunotherapies. Indeed, understanding the multifaceted landscape of the immune TME is essential to develop breakthrough strategies for fighting malignant tumors. (By Xueyan Mao and Xiaoran Yin).

The compilation of ten manuscripts delves into the Research Topic of “*Prediction and Evaluation of Tumor Immune Microenvironment Topographies*.” The main objective of this research is to enhance our comprehension of how to create a topographic map for the immune TME. The investigators discussed the pending issues associated with this ground-breaking field including basic research findings to novel computational approaches, clinic case reports, and research summaries. We aim to open potential translational venues and novel platforms for exploring the mystery of the therapeutic effects and adverse events (AEs) of tumor immunotherapy. This Research Topic includes (i) comprehensive reviews on biomarkers utilized for predicting patient response and prognosis when undergoing immunotherapies; (ii) illuminating viewpoints on examining the mechanism of the immune landscape associated with tumors in the TME; (iii) clinical case summaries and reports detailing the therapeutic outcomes and AEs of various forms of tumor immunotherapies; and (iv) original research on creating prognostic models with cutting-edge computational technologies based on clinical characteristics and big data of patients for predicting the responses and side effects of immunotherapies.

Notably, immunotherapy has achieved remarkable advancements through various treatment modalities, including immune checkpoint inhibitors (ICIs), cytokines, tumor vaccines, and cellular therapies, among others. Among all these approaches ICIs have demonstrated remarkable success in the treatment of different types of malignant tumors. Recent studies have unveiled the promising clinical efficacy of perioperative immunotherapy involving ICIs. Precise classification of perioperative treatment strategies based on their specific implementation methods is of paramount importance. Peng et al. conducted an extensive investigation into neoadjuvant therapies and biomarkers to identify the optimal approach to perioperative immunotherapy for non-small cell lung cancer (NSCLC). Their findings highlight that patients receiving a combination of immunotherapy and chemotherapy during the perioperative phase achieved enhanced outcomes with the favorable safety profile. Furthermore, for the optimization of patient outcomes, clinicians should meticulously consider the ideal time interval between neoadjuvant immunotherapy and surgical intervention. Despite significant progress in biomarker research, further in-depth exploration is imperative to refine personalized selection criteria for perioperative immunotherapy.

The effectiveness of immunotherapy is intricately linked to various factors pertaining to both tumor cells and the TME. However, our understanding of the interactions between tumor-infiltrating immune cells (TIICs) and tumors, as well as tumor cells’ influence on shaping the TME, remains elusive. The primary challenge lies in eliciting a robust anti-tumor immune response within a conducive TME, while overcoming the barriers of immunosuppression and immune exclusion. Consequently, the reconfiguration of the immune TME emerges as a viable strategy. Martínez et al. conducted an investigation into TME treatment, focusing on rectal cancer patients who underwent various neoadjuvant therapies, including chemotherapy, chemoradiotherapy (RCT), long-interval radiotherapy (LRT), and short-interval radiotherapy (SRT). Their research unveiled that patients with PIK3CA-mutated tumors exhibited elevated infiltration rates of tumor dendritic cells (DCs). Moreover, in comparison to conventional radiotherapy, LRT engendered a higher presence of interstitial helper T cells within the TME. Furthermore, the study observed that, relative to LRT, RCT resulted in diminished numbers of interstitial B cells and stromal T helper cells (Ths), regulatory T cells (Tregs), and cytotoxic T cells (CTLs), alongside an increased presence of swollen DCs in TME. Additionally, it was noted that local therapies such as radiotherapy elicited a more consistent overall immune response in comparison to no treatment or chemotherapy alone. These findings underscore the importance of discerning the divergent immune landscape induced by specific treatments, facilitating more individualized treatment decisions for rectal cancer patients in the future.

Metabolism research have provided invaluable insights into the intricate biological intricacies and the challenges associated with tumor immunotherapy. Several chemical compounds have been identified as potential targets for immunotherapy due to their influence on the progression of tumors within the immune system. One particular promising avenue of investigation is the dysregulated polyamine metabolism. Polyamines have emerged as key players in modulating the behavior of immune cells to mount an effective anti-tumor response, positioning them as compelling candidate for anti-tumor treatment. However, it is worth noting that polyamines may also exert immunosuppressive effects, promoting the development and progression of tumor cells by aiding them in evading immune surveillance. In a comprehensive study conducted by Lian et al., the effects of polyamines on the immune TME were meticulously examined, revealing a two-fold regulatory impact on both tumor cells and immune cells. This research unveiled that inhibiting polyamines can effectively reshape the immune landscape of TME. Furthermore, the findings from this study suggest that polyamines play a pivotal role in immunomodulation, contributing to tumor evasion. Consequently, polyamines are emerging as a promising avenue for advancing tumor immunotherapy. In a study by Liu et al., it was demonstrated that polyamines have a detrimental effect on the activity of DCs, NKTs, CD8^+^ TILs, and Th1 cells, while concurrently enhancing the function of Treg cells. Notably, polyamines exhibit a dual role in the context of NKs. Despite a range of preclinical trials involving inhibitors of polyamine metabolism, the clinical applications remain somewhat restricted and require further investigation to unlock their full potential in anti-tumor therapy.

Precision immunotherapy for tumors relies on the analysis of each patient’s genetic biomarkers to select the most appropriate individualized therapeutic regimen. Advancements in data mining and digital modeling have enabled researchers to leverage big data platforms and integrate various genome, proteome, and metabolic data to construct visual models. These models are increasingly finding application in clinical settings, aiding in the evaluation of treatment effectiveness, potential side effects, and overall prognosis, thereby ensuring accurate and reliable treatment for malignant tumor patients. In the realm of anti-tumor therapy, prognostic models provide a critical role in guiding clinical decisions for patients undergoing different combined strategies. Liang et al. developed an immune-related gene (IRGPI), to assess the effectiveness of immunotherapy in colorectal carcinoma (CRC). The index consists of 11 genes derived from transcriptome datasets and clinical data, accurately predicting survival rates, characterizing the immune TME, and gauging sensitivity to immunotherapy in CRC patients. Validation of the IRGPI’s utility was further confirmed through the CRC mouse model. Additionally, accumulating evidence underscores the intricate interplay between various factors that profoundly orchestrate tumor fate. Methylthioadenosine (MTA) phosphorylase (MTAP), a key enzyme in the methionine synthesis pathway, plays a pivotal role. The accumulation of MTA selectively inhibits the activity of protein arginine methyltransferase 5 (PRMT5) enzyme, resulting in increased sensitivity to PRMT5 inhibition in MTAP-deficient tumors. Notably, MTAP is closely located to the tumor suppressor CDKN2A on chromosome 9p21, leading to frequent genomic alterations involving MTAP/CDKN2A co-occurrence. Xu et al. discovered a connection between MTAP/CDKN2A deficiency and sarcomatous dedifferentiation in renal cell carcinoma (RCC), a finding that can predict aggressive disease progression, poor prognosis, primary resistance to targeted therapy, and potential favorable responses to immune checkpoint blockade. In the future, novel therapeutic options, such as immunotherapies or synthetic PRMT5 inhibitors, may offer new hope to RCC patients with *MTAP/CDKN2A* ^MUT^.

In addition, cuproptosis refers to copper-induced cell death, often accompanied by heightened mitochondrial-dependent energy metabolism and an increase in reactive oxygen species (ROS). Research suggested that an excess of copper is associated with various types of malignant tumors. Genes associated with cuproptosis could impact the development, progression, and metastasis of cancerous cells. Cai et al. developed a signature based on 43 cuproptosis-related genes to predict immune cell infiltration and evaluate the efficacy of immune checkpoint blockade (ICB) for individual patients. The cuproptosis risk score derived from this signature indicated that patients with kidney renal clear cell carcinoma (KIRC) who had higher risk scores experienced worse survival outcomes. This finding holds significant value in predicting the prognosis and facilitating precision therapy. Similarly, Wang et al. identified two distinct cuproptosis regulatory subtypes in hepatocellular carcinoma (HCC), each exhibiting unique immune cell infiltration characteristics. Differential gene expression between the two cuproptosis clusters was used to develop a risk signature model. This model revealed that patients in the high-risk group exhibited increased levels of immune and stromal cell infiltration and had a poorer prognosis. The regulatory patterns of cuproptosis may significantly contribute to the diversity of immune cell infiltration, enabling the quantification of individual patient risk and offering novel avenues for personalized anti-tumor immunotherapy in HCC patients.

There are still lots of puzzles in managing AEs in immunotherapy of pan-tumors. Thus, the management of those AEs, such as treatment-related adverse events (trAEs) and immune-related adverse reactions (irAEs), can pose a significant challenge. In a recent meta-analysis led by Kou et al., 21 randomized controlled trials were scrutinized to evaluate the trAEs associated with ICIs. Their findings indicated promising results for various perioperative immunotherapy strategies, notably in patients with operable NSCLC. Nevertheless, the quest for optimal dosing regimens and biomarkers persists, with the aim of tailoring treatments to individual needs and enhancing clinical practice. Fortunately, most AEs can be reversed with appropriate interventions. However, some trAEs can be serious, underscoring the importance of monitoring and comprehending AEs of ICIs therapy for safety outcomes. Among the most prevalent AEs for ICIs are skin-related reactions, with Stevens-Johnson syndrome being a potentially life-threatening cutaneous reaction. A case study by Li et al. documented the experience of a 76-year-old male patient with poorly differentiated metastatic lung adenocarcinoma. Following 9 weeks of sintilimab exposure (3 doses) combined with paclitaxel liposome subsequent to CRT, the patient developed Stevens-Johnson syndrome, affecting the limbs, trunk, lips, and oral mucosa. Skin tissue biopsy revealed infiltration of CD4^+^ and CD8^+^ T lymphocytes. However, studies evaluating discontinuation and restart strategies based on efficacy and AEs have not yielded definitive answers. Additionally, determining the optimal duration of ICIs treatment remains an ongoing challenge. The most recent research on customizing the length of immune checkpoint inhibitors (ICIs), as outlined by Yin et al., suggests ICIs tailoring treatment duration of according to an individual’s response patterns, tumor stage, and irAEs. This approach incorporates key assessments such as PET-CT, liquid biopsy (e.g., ctDNA), or tissue biopsy to guide discontinuation decisions. Moreover, ongoing prospective studies are expected to offer additional insights into the ideal duration of ICIs in the coming years.

Immunotherapy, an epoch-defining treatment, has revolutionized the way for the treatment of malignant tumors. It has significantly enhanced treatment efficacy, prognosis, and tolerability when compared to traditional therapies, providing new hope for patients. A crucial aspect of this approach involves analyzing the TME. The scientific insights gained from this field have yielded a wealth of knowledge on how to predict treatment responses and minimize AEs, thereby providing invaluable guidance for individualized therapy. This dynamic has prompted the establishment of a dedicated platform, fostering the identification and evaluation of the most efficacious immunotherapies while prioritizing patient safety. Although there remains a considerable learning curve concerning optimal dosing and the identification of biomarkers, the potential benefits of personalized treatment approaches are genuinely exhilarating, offering renewed hope to those in need of effective anti-tumor therapeutic strategies.

## Author contributions

XY: Writing – review & editing, Supervision, Writing – original draft. YF: Writing – original draft. BZ: Writing – original draft. XM: Writing – original draft. SC: Writing – original draft. YW: Writing – review & editing. SF: Writing – review & editing.

